# Mechanism of Wound-Healing Activity of *Hippophae rhamnoides* L. Leaf Extract in Experimental Burns

**DOI:** 10.1093/ecam/nep189

**Published:** 2011-03-20

**Authors:** Nitin K. Upadhyay, Ratan Kumar, M. S. Siddiqui, Asheesh Gupta

**Affiliations:** ^1^Defence Institute of Physiology and Allied Science, DRDO, Delhi 110054, India; ^2^Department of Toxicology, Jamia Hamdard, Delhi 110062, India

## Abstract

The present investigation was undertaken to evaluate the healing efficacy of lyophilized aqueous leaf extract of Sea buckthorn (*Hippophae rhamnoides* L., family Elaeagnaceae) (SBT) and to explore its possible mechanism of action on experimental burn wounds in rats. The SBT extract, at various concentrations, was applied topically, twice daily for 7 days. Treatment with silver sulfadiazine (SSD) ointment was used as reference control. The most effective concentration of the extract was found to be 5.0% (w/w) for burn wound healing and this was further used for detailed study. The SBT-treated group showed faster reduction in wound area in comparison with control and SSD-treated groups. The topical application of SBT increased collagen synthesis and stabilization at the wound site, as evidenced by increase in hydroxyproline, hexosamine levels and up-regulated expression of collagen type-III. The histological examinations and matrix metalloproteinases (MMP-2 and -9) expression also confirmed the healing efficacy of SBT leaf extract. Furthermore, there was significant increase in levels of endogenous enzymatic and non-enzymatic antioxidants and decrease in lipid peroxide levels in SBT-treated burn wound granulation tissue. The SBT also promoted angiogenesis as evidenced by an *in vitro* chick chorioallantoic membrane model and *in vivo* up-regulated vascular endothelial growth factor (VEGF) expression. The SBT leaf extract had no cytotoxic effect on BHK-21 cell line. In conclusion, SBT aqueous leaf extract possesses significant healing potential in burn wounds and has a positive influence on the different phases of wound repair.

## 1. Introduction

The skin is one of the largest organs in the body that performs numerous vital functions including fluid homeostasis, thermoregulation, immunologic, neurosensory and metabolic functions. The skin also provides primary protection against infection by acting as a physical barrier. When this barrier is damaged, pathogens have a direct route to infiltrate the body, potentially resulting in infection. Cutaneous wound repair consists of an orderly progression of events that establish the integrity of the damaged tissue. The sequence of events that repairs the damage is categorized into three overlapping phases: inflammation, proliferation and tissue remodeling. The normal healing process can be impeded at any step along its path by a variety of factors that can contribute to impaired healing [1]. Impaired wound healing may be a consequence of pathologic states associated with diabetes, immune disorders, ischemia, venous stasis and injuries such as burn, frost-bite and gun-shot wounds [2].

Burns are one of the most common and devastating forms of trauma. Healing impairment in burn injury is characterized by increased free-radicals-mediated damage, delayed granulation tissue formation, reduced angiogenesis and decreased collagen reorganization leading to chronic wound healing. Burn management entails significant duration of hospital stay, expensive medication, multiple operative procedures and prolonged period of rehabilitation [3]. Topical anti-bacterial agents and disinfectants are good in protecting against infection, but the occurrence of allergic reactions and skin irritations to these agents reduces the rate of skin regeneration and increases the recovery time [4, 5]. However, recombinant growth factors and tissue-engineered wound dressings are highly expensive and beyond the reach of most of the patients in the economically developing and underdeveloped countries.

In alternative and complementary systems of medicines such as Ayurveda, Siddha, Amchi, Chinese and aromatherapy, plants are being used to combat several disease and pathological conditions. Herbal products seem to possess moderate efficacy with no or less toxicity and are less expensive as compared with synthetic drugs. Many plants and plants-derived products have been shown to possess potent wound-healing activity [2, 6–8]. *Hippophae rhamnoides* L. subspecies turkestanica (family Elaeagnaceae) commonly known as Sea buckthorn (SBT) is a branched and thorny nitrogen-fixing deciduous shrub, native to Europe and Asia. The plant has been used extensively in oriental traditional system of medicine for treatment of asthma, skin diseases, gastric ulcers and lung disorders. All parts of the plant are considered to be rich source of a large number of bioactive substances like flavonoids (isorhamnetin, quercetin, myricetin, kaempferol and their glycoside compounds), carotenoids (*α*, *β*, *δ*-carotene, lycopene), vitamins (C, E, K), tannins, triterpenes, glycerides of palmitic, stearic and oleic acids and some essential amino acids [9, 10]. SBT has been found to possess significant anti-oxidative, anti-microbial, anti-inflammatory, immunomodulatory, radio-protective, adaptogenic and tissue regenerative properties [9, 11–15]. The aim of the present study was to investigate the healing efficacy of lyophilized aqueous SBT leaf extract on full-thickness burn wounds in rats and dissect its possible mechanism of action.

## 2. Methods

### 2.1. Experimental Animals

Male Sprague-Dawley rats, each weighing 180 ± 20 g, from the animal colony of the DIPAS, Delhi, were used for this study. The animals were maintained under controlled environment in the Institute's animal house at 25 ± 1°C and 12-h light-dark cycle. The experiments were performed in accordance with the regulations specified by the Institute's Animal Ethical Committee and conform to the national guidelines on the care and use of laboratory animals, India.

### 2.2. Collection and Extraction of Plant Material

The leaves of SBT were collected from the hilly regions (at an altitude of 2500–4000 m) of the North-West Himalayas (the region lies between latitude 32–36° North and longitude 76–79° East) in the month of September 2006, where the plant grows widely under natural conditions. Plant material (Voucher specimen SBTL-2006) was characterized by Dr O. P. Chaurasia, an ethanobotanist at the Defence Institute of High Altitude Research (DIHAR), Leh, India.

Fresh leaves of SBT were cleaned and washed thoroughly with water and re-washed with distilled water. Washed fresh leaves were dried under shade in a clean, dust-free environment. The aqueous extract was prepared by soaking powdered dry leaves in distilled water (1 : 5 w/v) at room temperature (25 ± 1°C). After 24 h, the supernatant was decanted and the residue re-soaked in fresh distilled water. The process was repeated four times for complete extraction. The supernatants were pooled, filtered through muslin cloth and centrifuged at 5000 g, 4°C. The supernatants obtained after centrifugation were frozen at −20°C and then lyophilized in Heto lyophilizer (HITOSICC, Heto-Holten A/S, Denmark). Lyophilized powder of the SBT leaf aqueous extract was stored at −20°C in an airtight plastic container until further use (yield 13.7% w/w). The high-performance liquid chromatography (HPLC) fingerprinting of each batch of the extract was carried out and maintained throughout the experiment, to avoid batch-to-batch variation.

### 2.3. MTT Assay for Cytotoxicity

In this study, the [3-(4,5-dimethylthiazol-2-yl)-2,5-diphenyltetrazolium bromide] MTT assay was used to assess cytotoxicity caused by the SBT leaf extract. BHK-21 cell line was purchased from the National Center for Cell Science (NCCS), Pune, India. The cells were maintained at 37°C in an incubator with a humidified atmosphere of 5% CO_2_ and cultured in Glasgow's; minimum essential medium (GMEM) containing 10% heat-inactivated fetal calf serum (FCS), streptomycin (100 *μ*g mL^−1^) and neomycin sulfate (50 *μ*g mL^−1^). The BHK-21 cells were seeded on a 24-well plate at 1 × 10^5^ cells mL^−1^. Sixteen hours after plating, cells were treated with various concentrations of the SBT leaf extract (25, 50, 100, 200, 400 *μ*g mL^−1^) for 24 h. MTT stock solution (100 *μ*L; 5 mg mL^−1^) was then added and incubated for 4 h. The formazan crystals generated in each well were dissolved in 1 mL of acidified isopropanol and read spectrophotometrically at 570 nm [8].

### 2.4. In Vitro Chick Chorioallantoic Membrane Model

The chick chorioallantoic membrane (CAM) model was used to assess the angiogenic activity of SBT extract as described by Lobb et al. [16]. Nine-day-old fertilized chick eggs were selected and a small window of 1.0 cm^2^ made in the shell. The window was opened and a sterile disk of methylcellulose loaded with SBT extract was placed at the junction of two large vessels. The window was resealed by tape and the eggs were incubated at 37°C in a well-humidified chamber. After 72 h, eggs were opened and new blood vessel formation was observed and compared with the control eggs containing disks without SBT extract. The photographic images of CAM model were taken for quantitative morphometric analysis of the density of blood capillaries in terms of number of red pixel per unit areas using National Institutes of Health Image J software (v1.38) and AngioQunat software.

### 2.5. Burn Wound Model

The animals were anesthetized by intra-peritoneal (i.p.) injection of thiopentone (25 mg kg^−1^), the dorsal surface of the rat was shaved, and the underlying skin was cleaned with 70% ethanol. Full-thickness burn wound was created by using an aluminum metal rod (diameter 1.5 cm, melting point 660°C) heated to 85°C. The temperature of the metal rod was monitored with a fabricated digital computerized multimeter. Hot rod was exposed on the shaved area of the rat for 20 s, resting on its own weight of 30 g. No additional pressure was applied on the hand-leaded metal rod. Single burn wound was created on dorsal part of each rat. After 24 h, dead tissues were excised using sterile surgical blade [17]. Animals were allowed to recover from anesthesia and housed individually in sterile cages.

### 2.6. Experimental Design

In the preliminary screening, dose-response study of the aqueous leaf extract of SBT was performed to find out the optimal concentration. Different doses of the extract (2.5, 5.0, 7.5 and 10.0%; w/w) prepared in soft white petroleum jelly (S.D. Fine Chem, India) were applied topically and twice daily for 7 days in rats. Silver sulfadiazine (SSD) cream USP, 1.0% w/w (Ranbaxy Laboratories Ltd., Delhi, India), was used for treating reference controls. Control group received the vehicle alone in an identical manner. The most effective concentration of the SBT extract was found to be 5.0% for burn wound healing and this was further used for detailed study.

#### 2.6.1. Pro-Healing Parameters

The wound surface area was traced on a transparent paper and measured planimetrically [2]. The granulation tissue excised on eighth postwounding day was used to analyze the biochemical parameters: hydroxyproline, hexosamine and total protein contents [18–20].

#### 2.6.2. Antioxidant Analysis

A 10% homogenate of granulation tissue was made in 0.15 M KCl containing 5 mM EDTA. After homogenization, samples were sonicated and an aliquot was withdrawn for the estimation of reduced glutathione (GSH) [21]. In the remaining homogenate, triton X-100 was added at 0.1% (v/v). Samples were incubated at 4°C for 2.5 h. After incubation, samples were centrifuged at 4226 g, and the supernatant was used for the estimation of superoxide dismutase, catalase, glutathione-*S*-transferase activities and vitamin C content [22–25]. Malondialdehyde (MDA), a marker for lipid peroxidation, was measured by the method of Ohkawa et al. [26]. Protein in the tissue samples was determined by the method of Lowry et al. [20].

#### 2.6.3. Histological Studies and Morphometric Analysis

The granulation tissues were preserved in 10% neutral formalin. The tissue sections of 6-*μ*m thickness were stained with hematoxylin and eosin and observed for histological changes under light microscope. Masson's trichome staining for collagen was also performed on paraffin sections, followed by photomicrography. All morphometric parameters were recorded with Image Analyzer (Olympus Microscope BX61) by using image analyzing computer program (Image-Pro Plus 6.2). All histological sections were assessed through the center of the wounds to obtain maximum wound diameter. The measurements were taken three times by examining the slides in random sequence, blinded to treatment. The thickness of the newly formed epidermis was measured at 1-mm interval and the mean was calculated. The blood vessel density was evaluated by taking average number of cells in six high-power fields (60X objective), midway in the wound bed.

#### 2.6.4. Gelatin Zymography

Matrix metalloproteinases (MMPs) expression was studied in the granulation tissues by gelatin zymography assay [17]. Granulation tissue was homogenized with Tris buffer (saline 0.9%, Tris 0.05 M, Triton X-100 0.25% and CaCl_2_ 0.02 M) and centrifuged at 4226 g for 30 min. Tissue homogenate (50 *μ*g) was subjected to 10% SDS-PAGE containing 0.1% SDS and 1 g L^−1^ gelatin under non-reducing conditions without prior boiling. After electrophoresis, gels were washed in 2.5% Triton X-100 for 30 min to remove SDS and allow protein to renature and gels were then subsequently immersed in activity buffer (50 mM Tris-HCl, 5 mM CaCl_2,_ 0.2 M NaCl, 0.02% NaN_3_) for 16 h at 37°C. The gels were then stained with 0.25% Coomassie brilliant blue (CBR-250) in methanol, acetic acid and water (4 : 1 : 5) followed by destaining with methanol, acetic acid and water (4 : 1 : 5). Enzymatic activities were detected as clear bands of gelatin lysis against a blue background [17].

#### 2.6.5. SDS-PAGE and Western Immunoblotting

Wound tissues were chopped into small pieces and collected in Tris buffer (50 mM, pH 6.8) containing protease inhibitors, phenyl methyl sulfonyl fluoride (PMSF) and aprotonin (Sigma, St Louis, MO, USA) at 10 and 2 *μ*g mL^−1^, respectively. Tissues were homogenized in Polytron homogenizer (PT 3100, Kinematica AG, Littau-Lucerne, Switzerland) with four strokes of 15 s each in ice bath. After homogenization, the samples were spun at 3000 g at 4°C and the total protein in the supernatant was measured according to the method of Lowry et al. [20].

Analysis of total protein in the tissue samples was done by PAGE in 4% (v/v) stacking and 10% (v/v) separation polyacrylamide gels in the presence of SDS, followed by staining with CBR-250. The homogenized tissue samples mixed in a sample buffer containing 1% SDS, 2% 2-mercaptoethanol and 10% glycerol were heat reduced in a boiling water bath, whereas the gel and running buffer contained 0.1 and 0.2% SDS, respectively. The molecular weight was determined using standard protein markers (broad range 200–6.9 kDa, Bio-Rad, Hercules, CA, USA) [17].

After electrophoretic separation of tissue proteins by SDS-PAGE, the proteins were electro-transferred on to a polyvinylidene difluoride (PVDF) membrane. The membrane was blocked for 1 h in Tris-buffered saline with 0.1% Tween-20 (TBST, pH 7.5) containing 5% milk protein. After incubation with rabbit polyclonal primary antibody (VEGF, Santa Cruz Biotechnology, CA, USA) and mouse monoclonal primary antibody (Collagen type-III, Sigma) for 2 h, the blots were washed extensively with TBST. Primary antibodies were revealed via incubation with alkaline phosphatase-conjugated secondary antibody, goat-anti-rabbit and goat-anti-mouse (Santa Cruz Biotechnology), respectively for 1 h. The blots then were developed with 5-bromo-4-chloro-3-indolyl phosphate/nitro blue tetrazolium (BCIP/NBT) liquid substrate system (Sigma) [17].

### 2.7. Statistical Analysis

Data are expressed as mean ± SE, and statistical significance between experimental and control values was analyzed by one way ANOVA followed by Dunnett's test using Graph Pad Prism 2.01 (Graph Pad Software Inc., La Jolla, CA, USA). A *P*-value <.05 was considered statistically significant.

## 3. Results

### 3.1. In Vitro Cytotoxicity Assay

No cytotoxic effect was observed on BHK-21 cells, when incubated under control conditions up to 400 *μ*g mL^−1^ of SBT aqueous leaf extract.

### 3.2. Enhanced Angiogenesis Revealed by CAM Model

**Figure 1:**
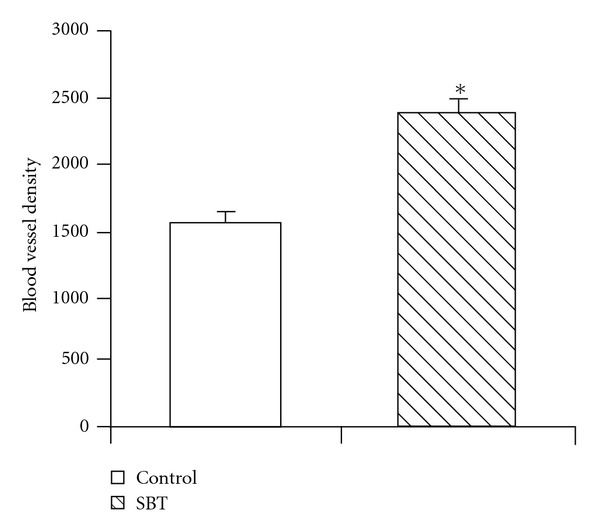
Density of blood vessels in *in vitro* CAM assays of chick eggs (12-days-old) for neovascularization. Control eggs were loaded with sterile methylcellulose disks and experimental eggs were treated with 80 *μ*g SBT leaf extract impregnated in methylcellulose disks. Blood vessels were analyzed in terms of number of red pixel per unit area using NIH Image J software (v1.38) and AngioQuant software. Values are mean ± SE of six eggs. *P < .05 compared with control untreated eggs.

In the *in vitro* CAM model for angiogenesis, methylcellulose disks containing 40, 80, 160, or 240 *μ*g SBT leaf extract were observed for new blood vessel formation. All the concentrations promoted angiogenesis but the maximum effect was observed with disk containing 80 *μ*g extract. Quantitative measurement of the CAM assay showed that relative density of arteries in the SBT treated eggs was much higher compared with eggs containing disks without the extract (Figure 1) (Supplementary Figure S1).

### 3.3. Wound-Healing Potential

Visual inspection of the wounds treated with SBT extract showed no evidence of wound bleeding, exudates, pus or wound inflammation at any time, and all wounds healed without incident. However, untreated burn wounds showed edema reflecting persistent inflammation, whereas wounds treated with SBT showed reduced or no edema. In general, the skin around the burns was soft and appeared normal except in the SSD group where it was dry.

#### 3.3.1. Augmented Pro-Healing Parameters

In dose–response study, it was observed that 5.0% (w/w) SBT leaf extract was most effective concentration for wound-healing activity.  Table 1 depicts the effect of topical application of various concentrations of SBT leaf extract on wound area reduction at different time intervals. The animals treated with 5.0% SBT extract showed significant faster reduction in wound area on day 4 (113.17 versus 138.5 mm^2^ in the untreated group) and day 8 (50.33 versus 90.83 mm^2^ in the untreated group) postwounding (Table 1). Levels of hydroxyproline, hexosamine, and total protein were also significantly increased in the rats treated with 5.0% SBT extract (Table 2).

#### 3.3.2. Antioxidant Status

Table 3 shows the antioxidants and MDA levels in the granulation tissue of control and SBT leaf extract (5.0%) treated burn wounds. There was significant increase in levels of enzymatic and non-enzymatic antioxidants such as glutathione (29%), superoxide dismutase (29%), catalase (23%), glutathione-*S*-transferase (20%) and vitamin C (46%), whereas the MDA level was significantly reduced (26%) in SBT treated burn wounds.

#### 3.3.3. Histological and Morphometric Examinations

**Figure 2:**
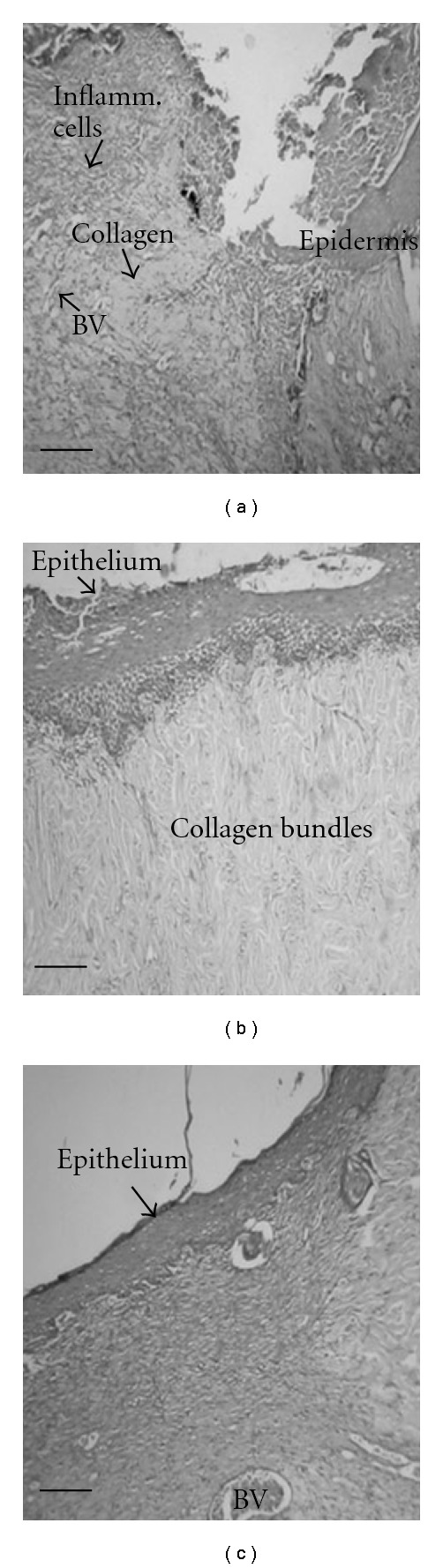
Histopathological changes on eighth post-wounding day in skin wound section of (a) control rats showing non-epithelialized wound surface with the presence of inflammatory cells, (b) SBT leaf extract (5.0%) and (c) SSD treated rats showing wound surface with well-organized thick epithelium. Collagen alignment is well developed in SBT leaf extract treated burn wounds. Scale bar, 100 *μ*m.

**Figure 3:**
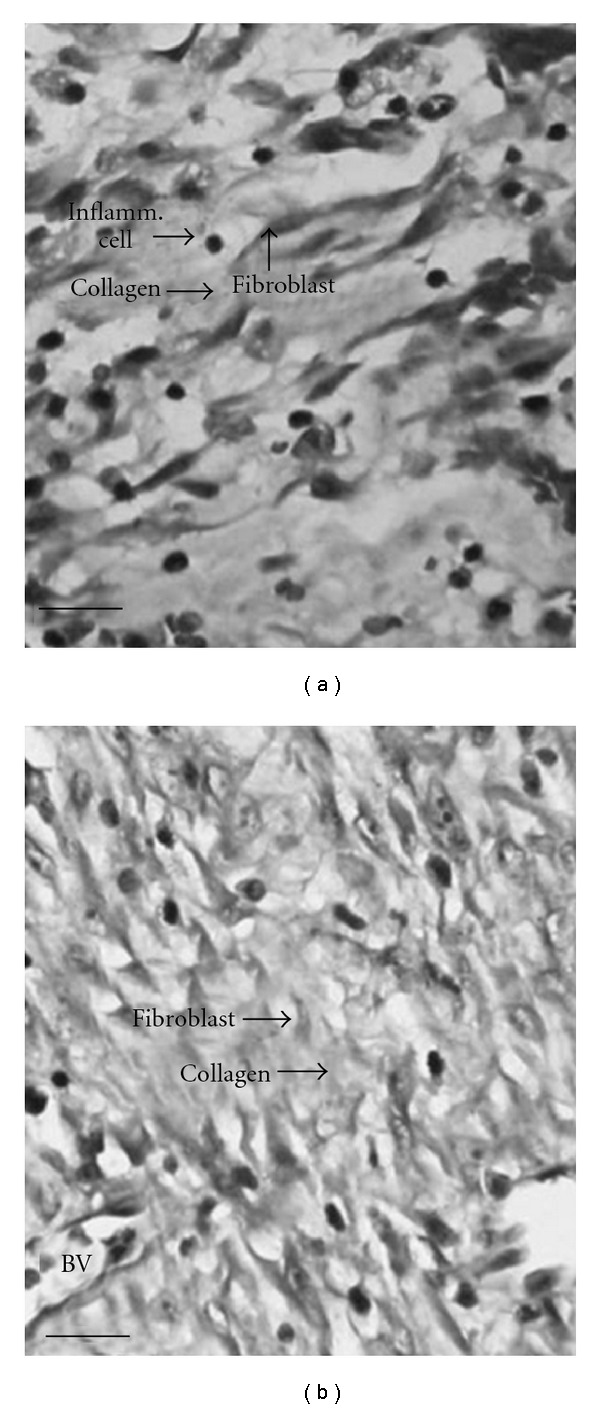
Masson's trichome staining for collagen on eighth-day postwounding in skin wound section of (a) control rats showing less and irregularly arranged collagen, (b) SBT leaf extract (5.0%) treated rats showing compact and well-aligned collagen fibers. Scale bar, 20 *μ*m.

On the day 8, the SBT treated group showed more advanced re-epithelialization and layering with continuous basement membrane in addition to a better organization of the collagen bundles. The SBT leaf extract and SSD treated animals showed reduced congestion, edema and polymorphonuclear leukocytes infiltration. The control group showed a distinct space between old and new regenerating layers of epithelium. The histological studies showed an overall early recovery and regeneration in the SBT treated group when compared with control group (Figure 2) (Supplementary Figure S2). The morphometric analyses of the histological sections showed that the SBT treatment resulted in increased blood-vessel density and thicker epidermis when compared with untreated burn wounds (Table 4). Furthermore, Masson's trichome staining showed uniform, compact and regularly arranged collagen fibers in the wound tissue of SBT treated rats, whereas untreated burn wounds had less compact and irregularly arranged collagen fibers (Figure 3) (Supplementary Figure S3).

#### 3.3.4. Enhanced Expression of MMPs

**Figure 4:**
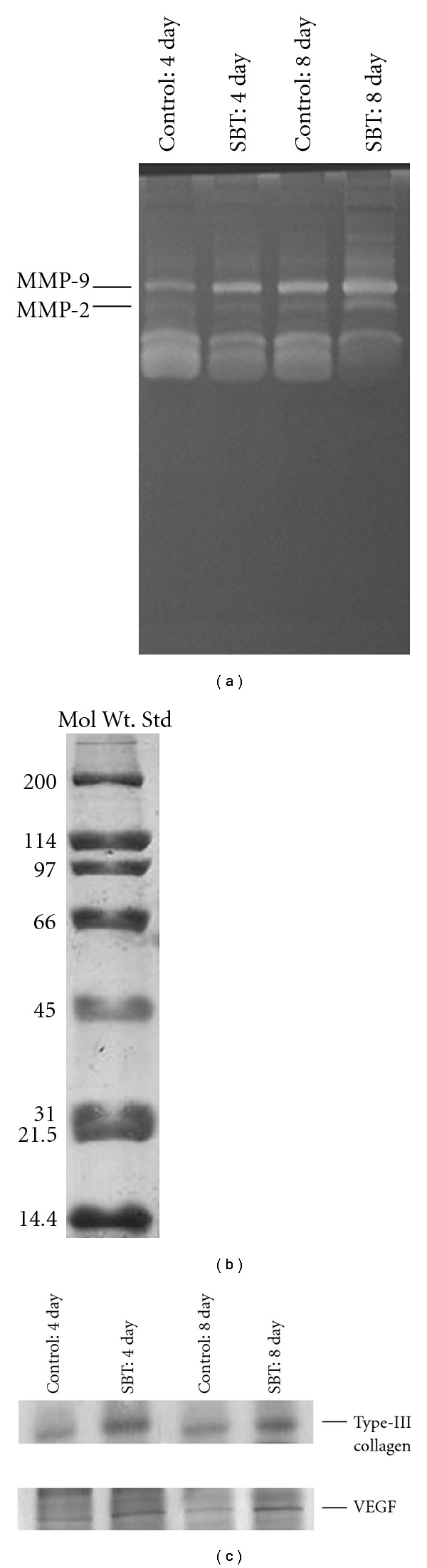
(a) Matrix metalloproteinase expression by gelatin zymography (10% SDS-PAGE, 1 g L^−1^ gelatin) in SBT leaf extract (5.0%) treated and untreated burn wound tissue of experimental rats after 4 and 7 days of treatment. (b) Expression of standard marker proteins (200–6.9 kDa, BIO RAD, 10% SDS–PAGE). (c) VEGF and collagen type-III analyzed by western blot in SBT leaf extract (5.0%) treated and untreated wound tissue of experimental rats after 4 and 7 days of topical treatment.

Gelatin zymography analysis of the granulation tissue after 4 and 7 days of topical treatment with SBT leaf extract showed increased expression of both MMP-2 and -9 in comparison with the control burn wounds (Figure 4(a)).

#### 3.3.5. Up-Regulation of VEGF and Collagen Type-III

SDS-PAGE analysis of granulation tissue on fourth and eighth day of treatment with SBT leaf extract in experimental rats showed differential expression of some proteins as compared with the control burn wounds. Western blot analysis showed an up-regulated expression of VEGF and collagen type-III in SBT treated wounds as compared with untreated control burn wounds (Figure 4(c)).

## 4. Discussion

The results of the present study indicated that the aqueous extract of SBT leaf promotes wound healing in experimental burn wounds. This was demonstrated by a significant increase in the rate of wound contraction, hydroxyproline, hexosamine and protein contents. The increased wound contraction in SBT treated rats might be due to an enhanced activity of fibroblasts in regenerated wound tissue. Myofibroblasts are believed to play a key role in wound contraction by exerting tension on the surrounding extracellular matrix (ECM) and secreting ECM proteins such as collagen to stabilize the contraction. Collagen is a major protein of ECM and component that ultimately contributes to wound strength [1]. The enhanced levels of hydroxyproline and hexosamine in SBT treated burn wounds probably provided the strength to the regenerated wound tissue. We observed enhanced expression of collagen type-III in SBT treated burn wounds as compared with untreated control group. Collagen type-III is a predominant form of collagen in the early stages of wound healing that helps in providing strength to the provisional ECM [1].

MMPs are key players in every phase of the healing process, that is, eliminate damaged protein, destroy the provisional ECM, facilitate migration to the center of the wound, remodel the granulation tissue, probably control angiogenesis and also regulate the activity of some growth factors [27]. Increased expression of MMP-2 and -9 in SBT treated experimental rats suggested that SBT played an important role in remodeling of the ECM.

Angiogenesis is a critical component of wound healing. Delayed or aberrant revascularization at the wound sites contributes to the etiology of chronic wounds [2]. The SBT treatment promoted angiogenesis in both *in vivo* and *in vitro* models as indicated by histological studies and new vessel formation in CAM model, which might be due to enhanced expression of VEGF in regenerated tissue. VEGF is an important pro-angiogenic cytokine and improves angiogenesis during wound healing by stimulating the migration and proliferation of endothelial cells through the ECM [28]. Furthermore, it has been reported that SBT extract significantly increases blood flow to the surrounding skin of the burn wounds and can enhance wound healing [29].

Free radicals are generated at the site of injury, which are known to impair the healing process by causing damage to cellular membranes, nucleotides, proteins and lipids [3]. In the present study, the MDA levels were found to be significantly decreased after SBT extract treatment, suggesting decreased oxidative injury, which could be due to increased quenching or scavenging of oxygen free radicals by the elevated levels of enzymatic and non-enzymatic antioxidants. The use of antioxidants has been shown to promote wound healing process [30]. The SBT leaf extract was found to be rich in flavonoids (catechin, rutin, quercetin, kaempferol and isorhamnetin) [10]. Flavonoids are efficient antioxidants, capable of scavenging free radical species and reported to play an important role in augmenting the wound-healing process [31–33]. Presence of flavonoids could be one of the factors contributing to wound-healing potential of SBT leaf extract.

SSD undoubtedly is a very efficient anti-bacterial agent; however, it is reported to have an inhibitory effect on the proliferation of fibroblasts and keratinocytes that may impair the wound-healing process [5]. In the present study, it was observed that prolonged application of SSD resulted in the excessive dryness of the regenerated and normal skin surrounding the burn area. However, the SBT treated wounds had softer surrounding skin and satisfactory regenerated wound tissue.

**Figure 5:**
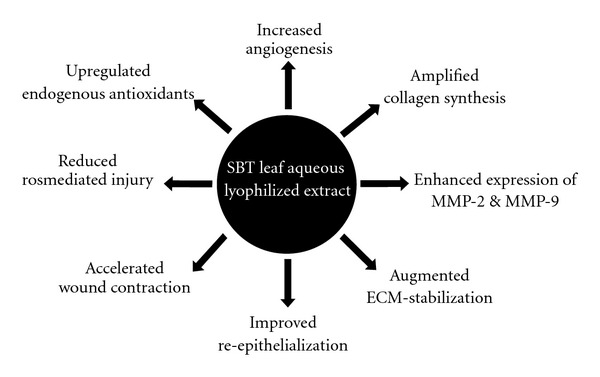
The schematic diagram showing the possible effect of the SBT leaf extract in promoting wound-healing activity.

In conclusion, as illustrated in Figure 5, the present study suggests that lyophilized aqueous extract of SBT leaves possesses a significant wound-healing activity in full-thickness burn wounds. The topical treatment with SBT leaf extract augmented endogenous antioxidants and prevented the free-radical-mediated tissue injury. It also played an important role in angiogenesis, ECM deposition and remodeling phase of wound healing.

## Supplementary Data

Supplementary Data are available at *ECAM* online.

## Funding

Defence Research and Development Organization, Delhi, India; Senior Research Fellowship from Council of Scientific and Industrial Research (CSIR), New Delhi, India (to N.K.U.).

## Supplementary Material

Figure S1: Relative density of arteries in the SBT treated eggs was much higher compared with eggs containing disks without the extract.
Figure S2: The histological studies showed an overall early recovery and regeneration in the SBT treated group when compared with control group.
Figure S3: Masson's trichome staining showed uniform, compact and regularly arranged collagen fibers in the wound tissue of SBT treated rats.

## Figures and Tables

**Table 1:** Effect of topical application of SBT lyophilized aqueous leaf extract on wound area contraction (mm^2^).

Post wounding day	Burn control	SBT aqueous leaf extract				SSD
		2.5% (w/w)	5.0% (w/w)	7.5% (w/w)	10.0% (w/w)
Day 0	176.83 ± 0.87	177.67 ± 1.26	178.33 ± 0.99	179.17 ± 0.60	177.33 ± 0.99	176.5 ± 1.80
Day 4	138.50 ± 4.60 (22)	124.50 ± 2.79 (30)	113.17 ± 3.48* (37)	121.33 ± 3.57* (32)	133.00 ± 3.17 (25)	128.83 ± 5.28 (27)
Day 8	90.83 ± 2.63 (49)	71.67 ± 3.68* (60)	50.33 ± 3.84* (71)	63.33 ± 3.79*(65)	79.00 ± 3.48 (55)	72.00 ± 4.33* (59)

Values are mean ± SE; *n* = 6; Numbers in parenthesis indicate percentage of wound contraction.

*P < .05 compared with burn control.

**Table 2:** Effect of topical application of SBT lyophilized aqueous leaf extract for 7 days on various biochemical parameters in granulation tissue.

Group	Burn control	SBT aqueous leaf extract				SSD
		2.5% w/w	5.0% w/w	7.5% w/w	10.0% w/w
Hydroxyproline (mg g^−1^ tissue wt.)	22.80 ± 0.32	29.12 ± 0.70*	29.96 ± 0.82*	26.72 ± 0.73*	23.19 ± 0.80	25.73 ± 0.67*
Hexosamine (mg g^−1^ tissue wt.)	0.50 ± 0.04	0.58 ± 0.03	0.71 ± 0.04*	0.62 ± 0.02*	0.60 ± 0.03	0.53 ± 0.03
Protein (mg g^−1^ tissue wt.)	88.74 ± 4.18	115.63 ± 7.40*	120.87 ± 7.77*	109.11 ± 3.17*	97.51 ± 3.61	109.78 ± 4.16*

Value are mean ± SE; *n* = 6.

*P < .05 compared with burn control.

**Table 3:** Effect of topical application of SBT lyophilized aqueous leaf extract (5.0% w/w) for 7 days on the levels of antioxidants and lipid peroxide (in terms of MDA) in granulation tissue.

Parameters	Burn control	SBT leaf extract
Glutathione (*μ*g mg^−1^ protein)	1.68 ± 0.07	2.16 ± 0.09*
Vitamin C (*μ*g mg^−1^ protein)	2.46 ± 0.37	3.60 ± 0.27*
Superoxide dismutase (U mg^−1^ protein)	1.25 ± 0.07	1.61 ± 0.06*
Catalase (U mg^−1^ protein)	8.18 ± 0.35	10.03 ± 0.46*
Glutathione-*S*-transferase (U mg^−1^ protein)	2.04 ± 0.08	2.45 ± 0.15*
MDA (nmol mg^−1^ protein)	2.41 ± 0.12	1.79 ± 0.21*

Values are mean ± SE; *n* = 6.

*P < .05 compared with burn control.

**Table 4:** Effect of topical application of SBT lyophilized aqueous leaf extract (5.0% w/w) for 7 days on epidermal thickness and blood vessel density.

	Burn control	SBT leaf extract	SSD
Epidermal thickness (*μ*m)	100.33 ± 5.86	149.83 ± 5.9*	147.83 ± 3.56*
Blood vessel density	1244.17 ± 32.91	2188.50 ± 119.60*	1278.50 ± 39.82

Values are mean ± SE; *n* = 6.

*P < .05 compared with burn control.
