# Supplementary material for: Mechanism of Wound-Healing Activity of Hippophae rhamnoides L. Leaf Extract in Experimental Burns
Source: Evid Based Complement Alternat Med. 2011 Mar 20;2011:659705. doi: 10.1093/ecam/nep189 (PMC3152935; doi:10.1093/ecam/nep189)
Supplement: Supplementary file 1 — Figure S1: Relative density of arteries in the SBT treated eggs was much higher compared with eggs containing disks without the extract. Figure S2: The histological studies showed an overall early recovery and regeneration in the SBT treated group when compared with control group. Figure S3: Masson's trichome staining showed uniform, compact and regularly arranged collagen fibers in the wound tissue of SBT treated rats. [file 659705.f1.pdf]

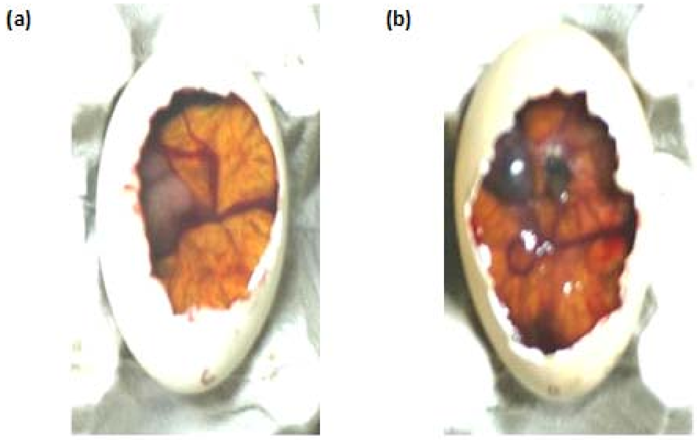

Figure 1S

Figure 1S. Colour photomicrographs of chorioallantoic membrane of chick eggs (12 day old) (a) showing vessel formation without SBT leaf extract treatment. Eggs were loaded with sterile methylcellulose disks. (b) showing increased number of vessels after treatment with 80 µg SBT leaf extract impregnated in methylcellulose disks. SBT, sea buckthorn.
