# Supplementary material for: Mechanism of Wound-Healing Activity of Hippophae rhamnoides L. Leaf Extract in Experimental Burns
Source: Evid Based Complement Alternat Med. 2011 Mar 20;2011:659705. doi: 10.1093/ecam/nep189 (PMC3152935; doi:10.1093/ecam/nep189)
Supplement: Supplementary file 2 [file 659705.f2.pdf]

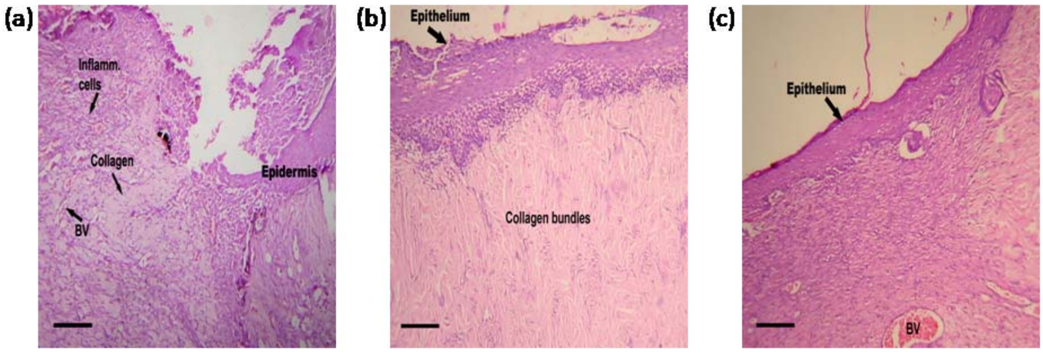


Figure 2

Figure 2S. Colour photomicrographs of Hematoxylin and eosin staining of the skin wound section of (a) untreated burn control rats on eighth post-wound day showing non-epithelialized (⟵) wound surface with slight edema and congestion. Skin wound section of SBT leaf extract (b) and silver sulfadiazine (c) treated burn wounds showing wound surface with well-organized thick epithelium (⟵). Neovascularization (BV) and collagen alignment is well developed in SBT leaf extract treated burn wounds. Scale bar 100 µm. SBT, sea buckthorn.
