# Supplementary material for: Mechanism of Wound-Healing Activity of Hippophae rhamnoides L. Leaf Extract in Experimental Burns
Source: Evid Based Complement Alternat Med. 2011 Mar 20;2011:659705. doi: 10.1093/ecam/nep189 (PMC3152935; doi:10.1093/ecam/nep189)
Supplement: Supplementary file 3 [file 659705.f3.pdf]

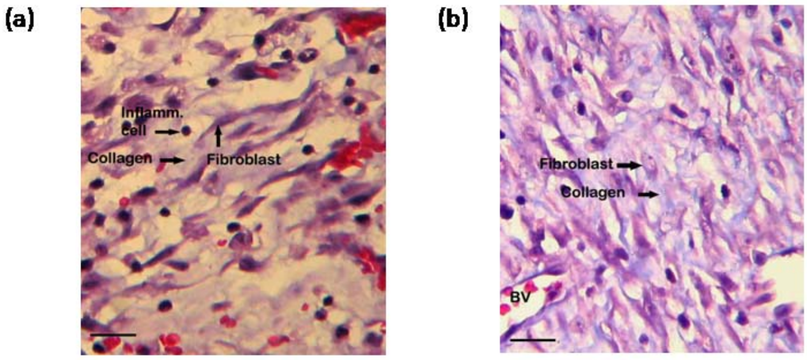

Figure 3

Figure 3S. Colour photomicrographs of Masson’s trichrome staining for collagen on eighth-day postwounding in skin wound section of (A) untreated burn control showing less and irregularly arranged collagen, (B) burn wounds treated with SBT leaf extract showing compact and well-aligned collagen fibers, (C) burn wounds treated with silver sulfadiazine. Arrows mark the collagen fibers. Scale bar, 20 µm. SBT, sea buckthorn
